# Automated video tracking of thrips behavior to assess host-plant resistance in multiple parallel two-choice setups

**DOI:** 10.1186/s13007-016-0102-1

**Published:** 2016-01-18

**Authors:** Manus P. M. Thoen, Karen J. Kloth, Gerrie L. Wiegers, Olga E. Krips, Lucas P. J. J. Noldus, Marcel Dicke, Maarten A. Jongsma

**Affiliations:** Laboratory of Entomology, Wageningen University, P.O. Box 16, 6700 AA Wageningen, The Netherlands; Laboratory of Plant Physiology, Wageningen University, P.O. Box 658, 6700 AR Wageningen, The Netherlands; PRI-Bioscience, Wageningen University and Research Center, P.O. Box 16, 6700 AA Wageningen, The Netherlands; Noldus Information Technology B.V., P.O. Box 268, 6700 AG Wageningen, The Netherlands

**Keywords:** Arabidopsis, Automated video tracking, Host-plant resistance, Western flower thrips, High-throughput phenotyping

## Abstract

**Background:**

Piercing-sucking insects cause severe damage in crops. Breeding for host-plant resistance can significantly reduce the yield losses caused by these insects, but host-plant resistance is a complex trait that is difficult to phenotype quickly and reliably. Current phenotyping methods mainly focus on labor-intensive and time-consuming end-point measurements of plant fitness. Characterizing insect behavior as a proxy for host-plant resistance could be a promising time-saving alternative to end-point measurements.

**Results:**

We present a phenotyping platform that allows screening for host-plant resistance against Western flower thrips (WFT, *Frankliniella occidentalis* (Pergande)) in a parallel two-choice setup using automated video tracking of thrips behavior. The platform was used to establish host-plant preference of WFT with a large plant population of 345 wild Arabidopsis accessions and the method was optimized with two extreme accessions from this population that differed in resistance towards WFT. To this end, the behavior of 88 WFT individuals was simultaneously tracked in 88 parallel two-choice arenas during 8 h. Host-plant preference of WFT was established both by the time thrips spent on either accession and various behavioral parameters related to movement (searching) and non-movement (feeding) events.

**Conclusion:**

In comparison to 6-day end-point choice assays with whole plants or detached leaves, the automated video-tracking choice assay developed here delivered similar results, but with higher time- and resource efficiency. This method can therefore be a reliable and effective high throughput phenotyping tool to assess host-plant resistance to thrips in large plant populations.

**Electronic supplementary material:**

The online version of this article (doi:10.1186/s13007-016-0102-1) contains supplementary material, which is available to authorized users.

## Background

Next-generation sequencing provides genomic information on large plant populations at increasingly fast rates and with diminishing costs [1]. This genomic information is of most value when linked to phenotypic traits of interest. In order to find genes underlying these traits of interest, efficient phenotyping platforms are urgently needed to reliably link genomic information to phenotypic information. Recently, major platforms have been established to phenotype plant populations with metabolomics [2], proteomics [3], transcriptomics [4] and automated imaging techniques [5–7]. High-throughput phenotyping is also key for future fundamental and applied research on plant–insect interactions [8, 9]. Some progress has recently been made in phenotyping plant resistance to pest insects (e.g. hemipterans [10] and lepidopterans [11]). However, no efficient systems have been developed to study host-plant resistance to thrips.

Host-plant resistance is considered one of the cornerstones in integrated pest management (IPM) and can be defined as ‘the relative amount of heritable qualities possessed by the plant which influence the ultimate degree of damage done by the insect in the field’ [12]. Pinpointing these heritable qualities on the genomes of plants can greatly enhance the development of insect-resistant crops [13]. However, identifying these traits is not a straightforward task, considering the wide range of components that contribute to host selection, host acceptance, growth, and reproductive success of herbivorous insects. Relevant factors include plant color, olfactory cues, plant topology and morphology, primary and secondary metabolites, and combinations of these factors [14]. Furthermore, the different components that underlie host-plant resistance are likely governed by multiple genetic loci, each marginally contributing in either positive or negative ways to the observed resistance. This complexity necessitates the development of reproducible high-throughput assays capable of dissecting the different components of host-plant resistance to insects [9].

Thrips are minute piercing-sucking insects and several species are major worldwide pests on vegetables and ornamental crops, especially due to their ability to act as vectors of tospoviruses [15]. Breeding for host-plant resistance to thrips is important for sustainable pest management, and of special urgency with species like Western flower thrips (*Frankliniella occidentalis*, WFT) that have become resistant to many pesticides [16]. Currently, methods to determine host-plant resistance to thrips fall into two broad categories: (1) ‘end-point assays’ monitoring plant damage and insect performance (reproduction and mortality) at the end of an experiment, and (2) behavior assays monitoring insect preference throughout the course of an experiment. End-point assays establish the quality of a host plant days or weeks post inoculation. In these assays the area of feeding damage on the plant caused by thrips is quantified or estimated. Thrips damage can be assessed manually [17], using imaging software [18], or, as recently described in field trials, using hyperspectral imaging [19]. In addition to damage assessments, insect performance (growth, survival and reproduction) can be recorded [17, 20]. Thrips performance has also been assessed in end-point assays that use plant extracts [17]. Behavioral assays include Y-tube olfactometers [21, 22] and flight tunnels [23] to establish the role of plant volatiles. Choice assays that manually record thrips settlement over time have been used to assess the role of non-volatile dietary deterrents. Some constitutive plant defense traits like protease inhibitors can take 6 h after ingestion before reaching their maximum effect on thrips behavior [24].

None of the above methods have been automated yet to allow parallel, unattended screening of variation in resistance traits to thrips. Characterizing thrips behavior as a proxy for host-plant resistance is a promising yet challenging alternative approach. Promising, because it allows detailed determination of the degree of host-plant acceptance over time; challenging, in relation to technical constraints that have to be solved, like the small size of these insects, their thigmotactic behavior (thrips tend to crawl underneath surfaces for cover) and their low contrast against the complex backgrounds of plant tissues.

This study describes automated video tracking of thrips behavior as a method to assess host-plant resistance, which can complement end-point analysis. Automated video tracking of animal behavior was introduced in the early 1990s [25, 26], but not applied until recently to the study of host-plant resistance to herbivorous insects [10]. Previously, we demonstrated the value of video tracking aphid behavior in non-choice assays, assessing host-plant resistance in *Arabidopsis thaliana* and lettuce against *Myzus persicae* and *Nasonovia ribisnigri* respectively [10]. Here, we present a behavior-based phenotyping approach using choice assays against a reference genotype. The method uses automated video tracking of thrips behavior in arrays of parallel two-choice arenas. No-choice assays may lead to traits involved in antibiosis (traits with toxic or antinutritive effects like allelochemicals or proteins), whereas choice assays may better expose traits involved in host plant preference and antixenosis (deterrent or repellent traits like antifeedants, volatiles, surface waxes) [8]. We applied this new phenotyping tool to screen 345 natural *Arabidopsis thaliana* accessions (the Arabidopsis HapMap population [27]) for thrips resistance, as compared to one reference accession (Col-0). This led to the identification of both highly susceptible and resistant Arabidopsis accessions. The video-tracking method was subsequently validated and optimized with two ‘extreme’ accessions from the HapMap population (Cur-3 and Rmx-A180).

## Results

### Prescreening the Arabidopsis HapMap population to identify accessions resistant and susceptible to Western flower thrips

**Fig. 1:**
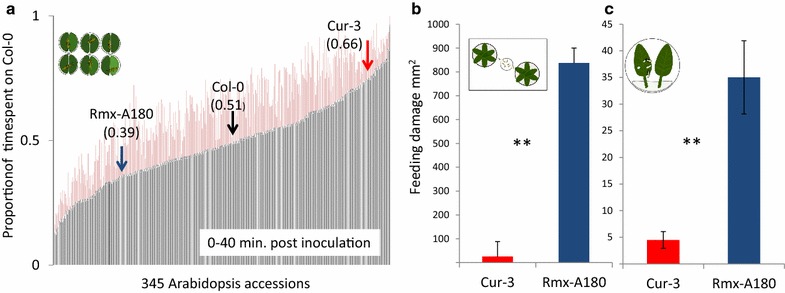
Phenotyping thrips resistance of *Arabidopsis* accessions with video tracking and damage assays. Thrips feeding preference was monitored with automated video tracking. Half leaf discs in a 96-well plate were used to screen the preference of thrips for 345 *Arabidopsis* accessions relative to reference accession Col-0. **a** The proportion of time thrips spent on Col-0 relative to the test accession is presented for 0–40 min post inoculation. Shown are genotypic mean ± SE (N = 5). **b** Feeding damage after 6 days, in a two-choice whole plant assay. Mean ± SE; N = 9, P = 0.004 (Wilcoxon signed-rank test, two-tailed). **c** Feeding damage after six days, in a two-choice detached-leaf assay. Two adult females were released in a Petri dish that contained one leaf of both lines. Mean ± SE; N = 24, P = 0.004 (Wilcoxon signed-rank test, two-tailed)

To identify Arabidopsis accessions that are resistant to WFT, thrips behaviour was monitored on 345 accessions in a preliminary video-tracking setup with a moderate throughput of 20 parallel assays. The video-tracking platform consisted of a stationary camera mounted above a 96-well plate illuminated from below, with fans to regulate temperature to minimize condensation (Additional file 1: Movie S1). Every well functioned as a two-choice arena by placing half a leaf-disc from a test accession and from a reference accession (Col-0) inside one well. Behavior of thrips on these two half leaf-discs was monitored to analyze host-plant resistance. In EthoVision XT, zone areas corresponding to the half leaf-discs of the control and test genotypes were assigned to quantify how much time thrips spent on either half in a recording period of 40 min. 345 accessions were screened in five separate rounds, using an incomplete block (alpha) design (see methods, statistics). Thrips resistance was measured as the proportion of time the thrips spent on the reference accession Col-0 compared to the test accession (Fig. 1a). In the most susceptible lines thrips spent less than 20 % of their time on the Col-0 reference accessions. In the presumably more resistant lines thrips spent on average more than 70 % of their time on the Col-0 reference. We selected one resistant (Cur-3) and one moderately susceptible (Rmx-A180) accession to confirm the difference in resistance to thrips in end-point feeding assays with whole leaves and whole plants.

### Comparing video tracking with end-point choice assays

To validate the resistant phenotype as selected by video tracking the HapMap population, the resistant Cur-3 and susceptible Rmx-A180 Arabidopsis accessions were screened as whole plants and detached leaves in two-choice assays for thrips resistance. For the whole plant assay, twenty adult female thrips were released in a closed container with one plant of either accession (N = 9). Feeding damage was assessed by counting the number of feeding spots. After six days, feeding damage was 33-fold higher on Rmx-A180 (P = 0.004, Wilcoxon signed rank test, Fig. 1b). The susceptibility of this accession relative to Cur-3 was also demonstrated by the 4-fold higher number of thrips offspring recorded on Rmx-A180 after six days (P < 0.0001) (Table 1). Manually assessing feeding damage on whole plants requires a lot of space and time and, therefore, detached leaf assays can be more practical. We, therefore, also carried out an end-point assay with detached leaves of Arabidopsis accessions in Petri dishes. Two adult females were released per Petri dish containing a detached leaf of both accessions. After six days five times more feeding damage was found on the susceptible Rmx-A180 accession (P < 0.001) (Table 1; Fig. 1c). Thus, the results of the automated video-tracking results were confirmed by these whole-plant and single-leaf endpoint assays.

**Table 1:** Results of three different dual-choice setups for testing thrips preference on two Arabidopsis accessions

Variable	Cur-3 resistant	Rmx-A180 susceptible
Video assay^a^ (total 8 h)		
Duration spent in zone (s)	5026 ± 470	8292 ± 631**
Duration not moving (s)	4122 ± 446	7494 ± 632***
Duration moving (s)	895 ± 80	787 ± 73*
Activity ratio (mov/not mov) (%)	22 ± 2 %	11 ± 2 %***
Distance moved (mm)	870 ± 70	926 ± 68
Movement velocity (mm/s)	0.65 ± 0.02	0.68 ± 0.02
Leaf assay^b^		
Damage after 6 days (mm^2^)	8.5 ± 2	45.6 ± 5***
Plant assay^c^		
Damage after 6 days (mm^2^)	25 ± 5.5	837 ± 62.3**
# of nymphs after 7 days	2.2 ± 0.2	8.7 ± 1***

* P < 0.05; ** P < 0.01; *** P < 0.001, Wilcoxon signed-rank test

^a^Behavior of thrips was monitored for eight hours (one adult female thrips per arena, N = 68, details of parameter settings in the methods section)

^b^Feeding damage was estimated after six days (two thrips per arena, N = 24)

^c^Feeding damage after six days was scored. In addition, the number of emerged nymphs was scored from the original inoculation of 20 adult female thrips per arena, N = 9). All assays used female adults of approximately 3 weeks old

### Method optimization

The method used to screen the HapMap population was limited in throughput (20 arenas) and duration (only a 40 min recording). The only parameter extracted in this initial thrips behavior screening was the proportion of time spent on the test accession compared to the reference accession Col-0. Our goals for optimizing the video-tracking platform were to (1) increase throughput with hardware adjustments (more arenas, better camera); (2) estimate phenotypic variance to accurately pin-point required replicates needed to perform these assays, (3) evaluate additional behavioral parameters (movement and non-movement). The two extreme lines used for these optimization steps were the resistant Cur-3, and susceptible Rmx-A180 (Fig. 1a, b). With a digital high-resolution camera we could track the behavior of thrips in 88 two-choice arenas simultaneously and recorded the behavior during eight hours with EthoVision XT. A demonstration movie of this setup with a sample of this recording can be viewed online (Additional file 1: Movie S1).

**Fig. 2:**
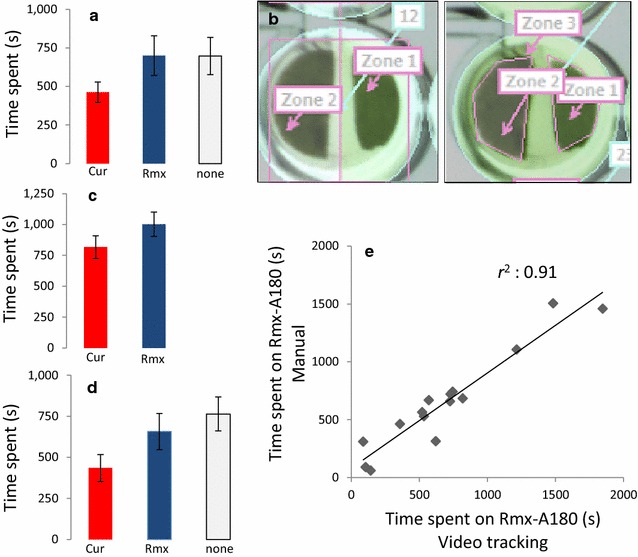
Validating arena settings with improved setup. Thrips behavior was assessed both visually and with automated video tracking in 15 arenas consisting of two-choice tests with Cur-3 versus Rmx-A180 Arabidopsis accessions. **a** Time thrips spent on either leaf disc, or on none of them (*circling around*, sitting on agar), based on manual annotation. **b** Arena settings used for initial HapMap population screening (*left panel*) and improved arena settings that manually highlight only the leaf discs, with a third zone referring to agar or boundary of the arena (*right panel*). **c** Automated video-tracking data of the same 15 arenas with initial arena settings. **d** Automated video tracking with improved arena settings. Mean ± SE; N = 15 **e** Correlation of scoring of the total time spent by thrips on accession Rmx-A180 with automated video tracking using EthoVision XT (X axis) and manual annotation using The Observer XT software (Y axis)

**Fig. 3:**
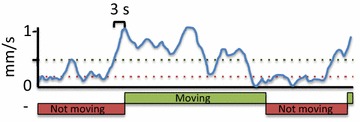
Movement determination in EthoVision XT. A movement event started when thrips obtained a speed above 0.5 mm/s averaged over 10 video frames (3 s) and stopped when speed dropped below 0.1 mm/s averaged over 10 video frames (3 s)

To validate the accuracy of the video-tracking method in annotating the correct location and behavior (movement and non-movement), we annotated the movement and location of thrips for 15 different arenas in this setup manually, using The Observer XT 10.5 software. This was done for 30 min of recording time and involved annotation of location (either Cur-3, Rmx-A180 leaf discs or elsewhere—agar, arena wall or cover) and movement status (moving or non-moving). The data show that in this first half hour of the analysis thrips were not recorded on any of the two leaf discs for 697 s on average (Fig. 2a). This accounts for roughly 37 % of the total recording, during which thrips either moved in circles in the upper part of the arena, or moved/rested on the agar. In the original arena settings used to screen the HapMap population, all arenas were divided in two zones. A zone referred to a leaf disc, as well as the surrounding area. To evaluate the effect of more accurate zone annotations, we applied zones that corresponded to the leaf outline exactly, and created a new zone that referred to all area that was not leaf (Fig. 2b). With the former “two-zone” settings, the time that thrips spent on either leaf disc was overestimated in comparison to manual annotation (Fig. 2c). By reshaping the zones individually to the leaf outline, we found distribution patterns that more accurately resembled the visual annotation (Fig. 2d). Manual annotations and video tracking correlated significantly, as exemplified for the annotated time thrips spent on the Rmx-A180 accession. (P < 0.001, Spearman Correlation test, Fig. 2e). Using these new settings, the correlation was higher than in the old settings (from *r*^*2*^ = 0.81 in old ‘two-zone’ settings to *r*^*2*^ = 0.91 in the new ‘three-zone’ settings). To evaluate differences in the time spent on feeding with automated video tracking, we used not-moving events as a proxy. Movement behavior was difficult to accurately score manually due to the relatively low resolution of the recordings. We therefore tried several movement settings in EthoVision XT and determined the optimal settings by visual inspection. We defined the start of a movement event as the moment when a subject was moving with a speed of >0.5 mm/s for at least 10 video frames (3 s), and this condition stopped when speed dropped to <0.1 mm/s for 3 s (Fig. 3).

### Thrips behavior on resistant versus susceptible accession

**Fig. 4:**
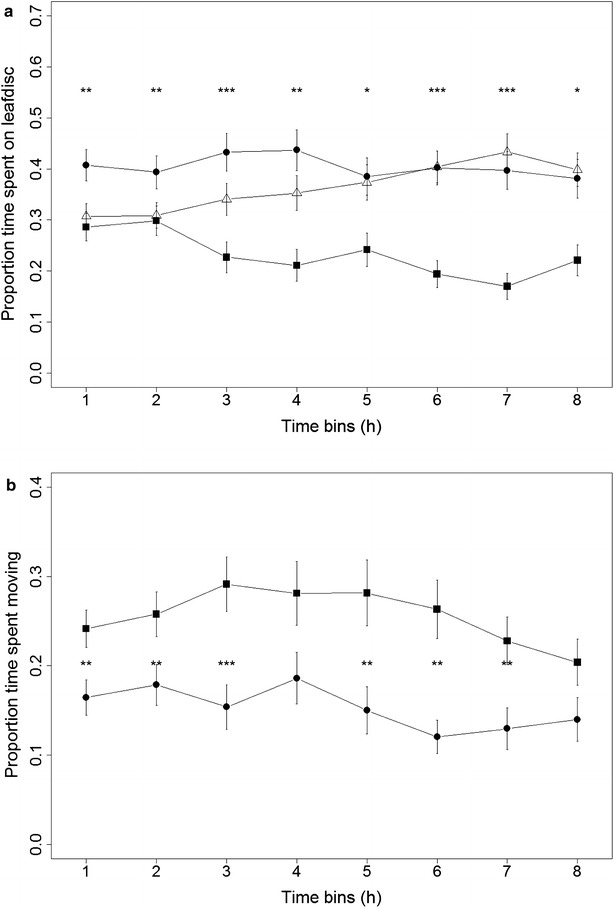
Thrips preference over time in two-choice test with Arabidopsis accessions Cur-3 versus Rmx-A180. **a** Two-choice assay with leaf discs of accessions Cur-3 versus Rmx-A180 showing the proportion of time spent by thrips on the resistant Cur-3 (*closed squares*), the susceptible Rmx-A180 (*closed circles*) and off leaf (*open triangles*). **b** Proportion of time spent moving on accessions Cur-3 (*squares*) and Rmx-A180 (*circles*). Mean ± SE, N = 68. (*P < 0.05, **P < 0.01, ***P < 0.001 Wilcoxon signed-rank test based on difference between the two accessions)

The entire Ethovision XT-recording of 88 arenas during 8 h was subsequently analyzed using the accurate zone annotation method. The analysis confirmed the results from the earlier 40 min behavioral observation that thrips spent significantly more time on the susceptible accession Rmx-A180, but this preference proved consistent now for the entire eight hours of recording (on average 61 % of time spent on any leaf was spent on accession Rmx-A180, P = 0.0012, Wilcoxon signed-rank test) (Table 1). Behavior can also be tracked over time in pre-defined time bins to study potential induced defenses, for instance. In assessing thrips behavior in time bins of one hour, we found a significant preference for the susceptible Rmx-A180 accession in all time bins, but 6 and 7 h post inoculation the largest difference was observed (Fig. 4a). Based on the movement settings described in Fig. 3, the total time spent not moving was found to be significantly longer on the susceptible Rmx-A180 accession, compared to the resistant Cur-3 accession (Table 1). The proportion of the total detected time spent moving differed significantly between the two accessions in all time bins, except at 4 and 8 h post inoculation (Fig. 4b).

### Throughput

The screening throughput for plant resistance to thrips strongly depends on the required replication level to achieve statistically significant results. In order to know the minimum number of replications for the genotype contrast of Cur-3 and Rmx-A180 we simulated experiments with different levels of replications and tested for significance. Simulated data sets (n = 10,000) were generated for video tracking and end-point assays, based on experimental means and standard deviations derived from this study. The generated data sets were subsampled with 1000 iterations without replacement for several replicate levels (n = 5, 10, 15, 20, 25, 30). Paired t-tests were executed for each iteration and the percentage of significant p-values (P < 0.05) per replicate level was calculated (Additional file 2: Table S1). The results simulate the efficiency to detect a degree of resistance as recorded for accession Cur-3 relative to a highly susceptible accession such as Rmx-A180 in the screening of large panels of different genotypes. Permutation tests on the video-tracking data set over 8 h showed that 15 replicates will lead to a majority of significant outcomes. Five replicates were enough to find significant differences among more than 50 % of the simulations in both end-point assays.

## Discussion

### Quantifying thrips behavior as a proxy for plant resistance

In this study, two-choice assays were used to assess host-plant resistance to thrips. With active insects like thrips, these two-choice assays generate activity distribution patterns on a test versus reference accession that are easy to obtain by means of automated video tracking. We used the proportion of time spent on a reference accession in our pre-screening of the Arabidopsis HapMap population to find resistant and susceptible accessions relative to Col-0. Our assumption was that leafdiscs of accessions on which thrips spend less time are more resistant. We validated this assumption by screening two extreme accessions from the HapMap population in several two-choice assays. The assumed resistant (Cur-3) and susceptible (Rmx-A180) accessions were confirmed to be resistant and susceptible respectively when screened against each other using the more detailed automated video tracking method on leaf discs and in two-choice end-point feeding damage assays on whole plants and detached leaves. The method of automated video tracking insect behavior in two-choice leaf disc assays was subsequently optimized using these two extreme Arabidopsis accessions from this population. For optimization, parameters relating to movement were quantified and analyzed. Movement time, distance and speed are parameters assumed to be associated mostly with searching for (better/more) food, whereas time spent not moving is assumed to be mostly associated with probing and feeding. Other confounding behavior activities like searching for shelter, grooming and resting will occur, but are assumed in the homogenous arena environment to represent systematic errors that are not genotype dependent. When thrips feed on plant cells, they thrust their stylets through the plant epidermis. This thrusting coincides with typical head nodding and could be an informative parameter to score as well [28]. However, the setup with a large number of 88 parallel arenas as used in this study was aimed at increasing throughput at the expense of resolution to monitor such detailed behavioral parameters. The recordings with Ethovision XT were done at the maximum available video resolution for live tracking (1280 × 960 resolution for a 100 × 75 mm area (1 mm^2^ = 164 pixels, pixel width 78 µm). A female adult WFT is approximately 1.4 mm long and 0.3 mm wide (0.42 mm^2^), which translates into approximately 69 pixels for one thrips in full view. Plant cell size is 10–100 µm and thrips may move only such distances between probes in neighboring groups of cells. Our movement threshold was set to <0.5 mm during 3 s. Consequently, one long non-movement event may refer to several feeding events in close proximity. Previous studies on cucumber plants showed that feeding scars from WFT on susceptible plants were grouped together, whereas the feedings scars on resistant plants were less numerous and more scattered. This correlated with more restless behavior and a bigger proportion of time spent walking [29] which would be picked up by our method of studying thrips behaviour. Previous studies on *Thrips tabaci* behavior showed that, in general, feeding was the predominant behavior recorded on leek and cucumber plants. *T. tabaci* were shown to spend roughly eightfold more time on feeding, than on inactivity [30]. The use of adult females that had been starved overnight before the experiment, was shown to make it even more likely that the ‘not moving’ state represents a feeding event. Starvation periods of at least four hours were shown to increase the response of thrips to visual and olfactory cues associated with a food source [23]. In our experiments using overnight-starved insects, we took the entire period of not moving as a proxy of time spent feeding assuming all non-feeding activities not to be genotype specific.

WFT were found to consistently spend 40 % less time on the resistant Cur-3 Arabidopsis accession, during the entire 8-h recording (P = 0.001, Wilcoxon signed-rank test). In addition, WFT spent significantly more time moving on the resistant Cur-3. Choice (genotype x/y) and activity (movement versus non-movement time per genotype) are independent behavioral parameters and both showed that thrips preferred the susceptible accession Rmx-A180, presumably spending more time feeding during the time of non-movement. Having several independent parameters to assess host-plant resistance to thrips is valuable in quantitative genetics. Different genes in plants might influence different aspects of thrips behavior. Although the duration of time spent in a specific zone showed a significant difference (choice parameter) for the two accessions tested, the highest levels of significance were found in the parameters “duration not moving” and “proportion of time moving”, indicating that the actual behavior exhibited on a specific leaf disc harbors variables that can be used to phenotype plants for thrips resistance more accurately. A potentially interesting next step would be to also discriminate between short and long non-movement events [10]. Short non-movement events could refer to test probes, non-movement events that last longer than 10 s are potential food uptake events. The Ethovision software version used could not yet produce statistics on individual events however.

### Advantages and limitations of automated video tracking

To evaluate advantages and limitations of the end-point assays and automated video-tracking assays used in this study, we listed the different variables that can be determined with these assays, the number of insects used per assay, the duration of one experiment in each assay, and the actual labor involved in performing these assays (Table 2). There are five main advantages of automated video tracking over end-point measurements:

**Table 2:** Comparison of three different two-choice assays to acquire data on plant resistance to thrips

	Whole plants	Detached leaves	Video tracking
			
Main advantages	Non-invasive Reproduction and survival data	Limited space and number of insects required More standardized setup (allows automated imaging)	Detailed behavioral parameters Quick and objective controlled conditions
Main disadvantages	Space consuming Large numbers of thrips required Time consuming analysis large environmental variation	Limited mechanical damage at petiole Senescence of material Time consuming analysis	Mechanical damage at edge of leaf disc Relationship to endpoint values unknown
Variables obtained	Feeding damage Reproduction	Feeding damage	Duration spent in zone Duration not moving Duration moving Ratio moving/not moving Distance moved Velocity
Inoculation	30 min	60 min	30 min
Duration	6 days	6 days	8 h
Analysis	2–4 h	1 h	10 min
Minimum number of replicates^a^	5	5	15
# thrips required	100 (5 × 20)	10 (5 × 2)	15 (15 × 1)

^a^The minimum number of replications is based on the criterion that >50 % of experiments should be significantly different among genotypes [P < 0.05 (Additional file 2: Table S1)]

1. More detailed choice and movement parameters relating to a specific developmental stage of the insects (adult, nymphs) on a specific developmental stage and tissue of a plant are obtained and these are made relevant using a direct within-assay comparison to a reference plant genotype and followed for as long as 8 h. These behavioral parameters on specific tissue samples can be the result of component traits such as leaf volatiles, leaf toughness, constitutive and induced chemical defenses that add up positively or negatively to the overall susceptibility/resistance of plants as measured by endpoint assessment. Dissecting overall plant defense into component traits, as done in video tracking, is expected to lead to stronger genetic signals in quantitative genetic studies [9].

2. The automated video-tracking method is faster and more objective than the current rating systems that visually score feeding damage, which often do not allow precise quantification and are sensitive to subjectivity and inconsistency of the human observer [8]. The automated process in which video-tracked thrips behavior is dissected into components of choice, movement and speed is not subject to human measurement and annotation errors and the data are much more quickly obtained and statistically analyzed. Permutation tests on the video-tracking data set over 8 h showed that 15 replicates will lead to a majority of significant outcomes. However, this is based on the experimental means of two accessions (Cur-3 and Rmx-A180) that are found at opposite extremes of the host-plant resistance spectrum. More replicates are likely necessary to pick up more subtle differences in resistance or tolerance.

3. Controlled conditions. The use of leaf discs with uniform plant biomass of a chosen tissue type and developmental stage in closed arenas immediately after harvesting for just a few hours removes a lot of plant and environmental variability accumulating during prolonged multiday experiments on whole plants or detached leaves. The validity of comparisons between independent experiments will be improved in this way. In addition, plant samples can be taken from their normal optimal production site like an open field which normally would not allow a proper insect resistance test to be performed.

4. In genetic studies there is often only one plant per genotype (e.g. from crossing populations, outcrossing species) which makes it far more difficult to efficiently identify genetic markers linked to insect resistance if whole plants or leaves are required for replication. In video tracking, multiple leaf discs can be generated from a single plant or leaf to obtain a practically reliable estimate of the resistance level for a particular plant genotype but not accounting for interplant and environmental variation.

5. The space and resource efficiency of the use of leaf discs is much greater. The 88 parallel experiments required an experimental space of only 100 cm^2^, requiring fewer insects than whole plant damage assays and only 1/6th or less of the time. In plant breeding, insect assays are mostly carried out in greenhouse compartments requiring thousands of insects with a lot of containment measures to avoid the spread of insects to other parts of the greenhouse. Here, the plants may be evaluated for other traits in parallel, can be grown in the open field, and only need to sacrifice a few leaves for tests done in the laboratory.

There are also downsides to the use of leaf discs. The generation of leaf discs introduces mechanical damage that may induce or inhibit physiological processes unrelated to thrips infestation and potential resistance under natural circumstances. It also offers only a narrow window on the total insect-plant interaction during plant and insect development. Yet, some of the issues raised against the use of leaf discs with phloem-feeding insects like whiteflies and aphids [31], are of less concern when working with epidermal cell-feeding insects like thrips which do not depend on phloem turgor for normal behavior. Our leaf-disc assays showed the same pronounced differences as the end-point assays with intact plants and detached leaves. In general, video tracking methods offer major advantages for prescreening a plant population, but resistance characteristics in selected genotypes should always be validated under field or greenhouse conditions to validate their relevance.

## Conclusion

End-point measurements and detailed initial behavioral screenings are essentially complementary approaches for measuring different aspects of insect resistance and potentially generate different outcomes. Combining these approaches will, therefore, be the most robust approach to efficiently identify the factors responsible for thrips preference and performance. The two-choice video-tracking platform presented here may proof to be a valuable high-throughput alternative to the classical damage assays to assess host-plant resistance to thrips. This method in its optimized form can screen hundreds of plant samples per set up per day. This will likely benefit selection and breeding of cultivars that are resistant to piercing-sucking insects.

## Methods

### Insects

The Western flower thrips (*Frankliniella occidentalis* (Pergande)) used in this study were originally collected from chrysanthemum flowers and reared on green common bean pods (*Phaseolus vulgaris*) in glass bottles placed in a climate chamber (25 ± 1 °C, L:D 8:16). Twice per week, 200 adult females were transferred to fresh bottles with bean pods to synchronize the offspring production. In the experiments adult females (20 days after emergence of larvae from the eggs) were used, that were starved overnight in Perspex tubular cages closed on one side with gauze and on the other side with two layers of stretched sheets of Parafilm containing a droplet of water to enable drinking. Thrips were anesthetized with CO_2_ and placed on ice just prior to experiments.

### Plants

We used *Arabidopsis thaliana* as host plant species. Initial screening of resistance to thrips was done for the HapMap population, consisting of Arabidopsis accessions collected globally [27]. We obtained phenotypic information on 345 out of the 360 available accessions. Rmx-A180 (CS76220, collected by J. Bergelson, latitude 42,036, longitude −86,511, Michigan, USA) and Cur-3 (CS76115, collected by F. Roux, latitude 45,000, longitude 1.75, France) were used for follow up experiments. For insect assays, plants were grown from seeds in small plastic pots (5 cm diameter) on pasteurized soil (4 h at 80 °C; Lentse potgrond, Lent, The Netherlands) in a climate room (21 ± 1 °C, 50–70 % relative humidity; 8 h:16 h L:D photperiod; light intensity 200 μmol m^−2^ s^−1^). For all experiments, 5-week-old plants were used.

### Video-tracking setup

Thrips behavior in the HapMap population screen was recorded with a monochrome camera (Ikegami, model: I CD-49E, type: REV, 768 × 576 pixels (PAL), analog output) with a varifocal lens (Computar H3Z4512 CS-IR, 4.5–12.5 mm F1.2) for the HapMap population screening. This allowed the screening of 20 two-choice arenas simultaneously. In the optimization step with two extreme Arabidopsis accessions, we used a digital camera (GigE Basler acA2040-25gc). In both cases, a backlight unit (FL tubes, 5000 K) was used to illuminate the arenas. Ca. 1 cm above the backlight unit, 96-wells microtiter plates (flat bottom suspension cells from Sarstedt, product number 831835500) that contained the two-choice arenas were placed on a custom made platform. A fan blew air between the backlight unit and microtiter plate to prevent condensation. Room temperature was kept constant at 21–22 °C.

### Video-tracking software settings

We tracked thrips behavior with EthoVision^®^ XT 10.0 (Noldus Information Technology B.V., Wageningen, The Netherlands) video tracking and analysis software. Due to the large number of arenas screened simultaneously (88), and the method to detect insects (live tracking), a maximum resolution of 1280 × 960 pixels with 3.5 video frames per second was used. Dynamic subtraction and center-point detection were used as detection methods, with a dark contrast of 8–255. Subject size detection was limited to the range of 10–160 pixels. Pixel smoothing was set to medium. Moving thresholds were set to start when thrips velocity reached above 0.5 mm/s averaged over 10 video frames (3 s) and stopped below 0.1 mm/s (Fig. 3).

### Arabidopsis HapMap population screening

Thrips preference was phenotyped in two-choice arenas using 96-well plates, consisting of two half leaf discs from Col-0 and one of the HapMap accessions. Arabidopsis leaf discs (6 mm in diameter) were punched with a cork borer, cut in half and placed into the wells with soft tweezers on a layer of 1 % technical agar that filled the wells for ¾ of the volume. Position bias was corrected for, by alternating the Col-0 leaf disc position (left or right) in every row. Female adult thrips (starved overnight) were anesthetized and kept on ice prior to the recordings. A soft brush was used to place thrips in the individual wells. Optical adhesive film (Micro Amp, Biosystems) was used to seal of the 96 well plates to prevent thrips from escaping. Plants were screened in 5 rounds of 360 accessions. Plants were randomly allocated to blocks (20 accessions per block, 18 blocks per round). One sampling day consisted of 5 blocks (100 accessions), except for the last day (3 blocks, 60 accessions). Manual quality checks on all recordings detected some arenas with non-moving thrips. These non-moving thrips were considered dead, and discarded from the analyses. Thrips position was monitored for 40 min with an analog monochrome camera mounted 50 cm above the two-choice arena plate. The proportion of time spent on accession Col-0 was assessed with EthoVision XT software and used as a proxy for host-plant preference.

### Video tracking of two extreme Arabidopsis accessions

Arabidopsis accessions Cur-3 (resistant) and Rmx-A180 (susceptible) were used for optimizing the video tracking setup. A digital camera (GigE Basler acA2040-25gc) allowed the screening of 88 arenas simultaneously. Arenas were set up the same way as the HapMap screening, except that in this assay a half leaf disc of each of the two extreme accessions were placed in one well and an additional neutral zone was created. Thrips behavior was monitored for 8 consecutive hours. In addition to the proportion of total time thrips spent on one accession (parameter used to assess resistance in the HapMap screening), additional parameters were “duration of time not moving (s)”, “duration of time moving (s)”, “movement proportion per genotype”, “distance moved (mm)” and “movement velocity (mm/s)”. A movement event started when thrips obtained a speed above 0.5 mm/s averaged over 10 video frames (3 s) and stopped when speed dropped below 0.1 mm/s averaged over 10 video frames (3 s). The Observer XT 10.5 Software (Noldus IT, Wageningen, The Netherlands) was used for visually assessing thrips position in 15 selected arenas for the first half hour of recording, to validate detection and arena settings in automated video tracking.

### Whole-plant assay

Plastic containers (length: 17 cm, width: 11.5 cm, height: 6.5 cm) functioned as two-choice whole plant arenas. The transparent lids of the containers had a circular piece of mesh in the center for ventilation. Thrips preference was screened in nine replicates and evaluated by placing both plants in opposite corners with a perspex tubular cage closed on one side with gauze in the middle that contained 20 adult female thrips (starved overnight) per treatment. The containers were then placed in a climate chamber (25 ± 1 °C, L:D 8:16). Feeding damage was estimated in mm^2^ after 6 days by counting the number of small feeding spots on the entire plants. One small spot accounted for approximately 3 mm^2^ damage (bigger spots were counted as 2–5 small spots). The number of adult and juvenile thrips on both plants was determined by submerging and shaking the aboveground tissues in a flask of 70 % ethanol and filtering it through a mesh. The residue of thrips was flushed from the filter into a Petri dish to count adult and juvenile thrips separately using a stereomicroscope.

### Detached-leaf assay

‘Thrips proof’ Petri dishes with a diameter of 5 cm (BD falcon, Product Number: 351006) were used for two-choice detached-leaf assay (N = 24). One ml of 1 % technical agar was poured into a Petri dish, that was left to solidify in a 20° slope. One leaf per plant was harvested with a pair of scissors, and the leaf petiole was inserted into the layer of 1 % technical agar, alternating left–right, to compensate for potential position effects. Two adult female thrips (3 weeks after egg hatching, starved overnight at 25 °C) were used. After 6 days a climate chamber (25 ± 1 °C, L:D 8:16), feeding damage was assessed as described above for the whole-plant assay.

### Statistics

Data distribution and homogeneity of variances of all two-choice assays were tested with a Shapiro test and a Levene’s test. Normally distributed data were tested with a paired Student’s *t* test, data that were not normally distributed were tested with Wilcoxon-signed rank tests using IBM SPSS Statistics 19 software. Correlation between manually annotated behavior of insects, and behavior annotated with automated video tracking was tested with a Pearson correlation test. For the screening of the HapMap population, plants were screened in 5 rounds (complete replicates) using an incomplete (alpha) block design for 360 accessions (phenotypic data were obtained only for 345 accessions). Within each round plants were randomly allocated to 18 blocks of 20 accessions, the blocks representing plants being screened in one recording. One sampling day consisted of 5 blocks (100 accessions), with the exception of the last day (3 blocks, 60 accessions). Genotypic means (BLUEs) were calculated using the following linear mixed model:

$${\text{Y}} =\upmu + {\text{REP}} + {\text{GEN}} + {\text{REP:BLOCK}} + {\text{E}},$$

where REP denotes complete replicate and REP:BLOCK is a random term for blocks nested within replicate. From the 360 accessions in the block design, we obtained phenotypic information for 345 accessions. Simulation to assess the number of required replicates were done in R, using the rnorm command. Simulated datasets were created (n = 10,000) for video tracking and end-point data, using the mean and STDEV derived from the experiments described in this study. The mean and STDEV values for Cur-3 and RMX-A180 to generate the simulated datasets were respectively 7926 ± 5252 and 12,159 ± 5610 (seconds spent on each leaf disc in 8 h of recording), 1352 ± 783 and 1890 ± 912 (seconds spent on each leaf disc in the first hour of recording), 8.5 ± 9.9 and 45.6 ± 24.4 (mm^2^ feeding damage detached leaf assay) and 837.7 ± 187.2 and 25.7 ± 16.5 (mm^2^ feeding damage whole plant assay). Figures 1 and 2 were created in Windows Excel 2010, Fig. 3 in PPT and Fig. 4 in R [32].

## Additional files

10.1186/s13007-016-0102-1 **Demonstration movie of automated video tracking thrips behavior in two-choice assays.** This movie shows the preparation of two-choice leaf disc assays with Arabidopsis, the camera setup and the actual tracking of Western Flower Thrips behavior with EthoVision XT video tracking software.
10.1186/s13007-016-0102-1 Simulation predicting the fraction of experiments yielding significant differences between Cur-3 and Rmx-A180 in relation to the number of assay replicates^1^.

## Abbreviation

WFT: Western flower thrips
