# Supplementary material for: Automated video tracking of thrips behavior to assess host-plant resistance in multiple parallel two-choice setups
Source: Plant Methods. 2016 Jan 18;12:1. doi: 10.1186/s13007-016-0102-1 (PMC4717623; doi:10.1186/s13007-016-0102-1)
Supplement: Supplementary file 2 — 10.1186/s13007-016-0102-1 Simulation predicting the fraction of experiments yielding significant differences between Cur-3 and Rmx-A180 in relation to the number of assay replicates1. [file 13007_2016_102_MOESM2_ESM.docx]

| **Table S1. Simulation predicting the fraction of experiments yielding significant differences between Cur-3 and Rmx-A180 in relation to the number of assay replicates^1^**   \| **# replicates** \| **5** \| **10** \| **15** \| **20** \| **25** \| **30** \| \| --- \| --- \| --- \| --- \| --- \| --- \| --- \| \| **behavior assay (1 hour)** \| 0.17 \| 0.36 \| 0.53 \| 0.64 \| 0.75 \| 0.83 \| \| **behavior assay (8 hours)** \| 0.12 \| 0.23 \| 0.35 \| 0.46 \| 0.55 \| 0.64 \| \| **leaf assay** \| 0.67 \| 0.98 \| 1.00 \| 1.00 \| 1.00 \| 1.00 \| \| **whole plant assay** \| 1.00 \| 1.00 \| 1.00 \| 1.00 \| 1.00 \| 1.00 \| |
| --- | --- | --- | --- | --- | --- | --- | --- | --- | --- | --- | --- | --- | --- | --- | --- | --- | --- | --- | --- | --- | --- | --- | --- | --- | --- | --- | --- | --- | --- | --- | --- | --- | --- | --- | --- |
| **^1^**Significant differences (P < 0.05, paired t-test, based on simulated data (N=10,000). Simulated data sets were generated to estimate the required replicates necessary for video tracking and end-point assays, based on experimental means and standard deviations derived from this study. The generated data sets were subsampled with 1000 iterations without replacement for several replicate levels (n = 5, 10, 15, 20, 25, 30). Paired t-tests were executed for each iteration and the percentage of significant p-values per replicate level was calculated. The results simulate the efficiency to detect a degree of resistance as recorded for accession Cur-3 relative to a highly susceptible accession such as Rmx-A180 in the screening of large panels of different genotypes. |
